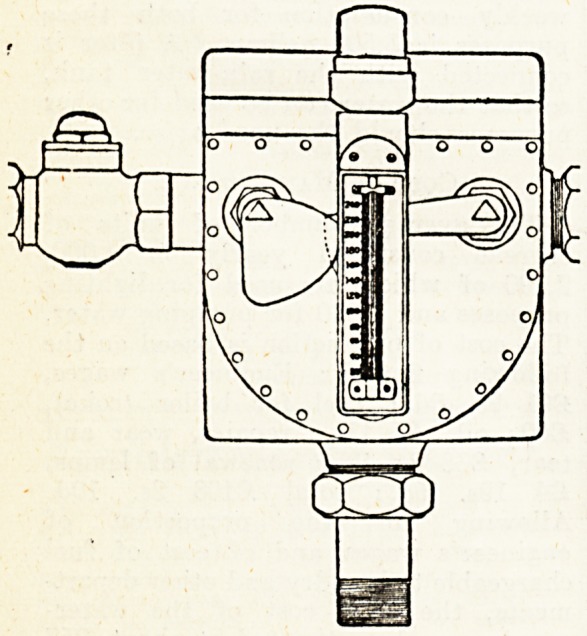# The Institutional Worker

**Published:** 1913-06-14

**Authors:** 


THE
### INSTITUTIONAL WORKER
**Being a Special Supplement to "The Hospital"**

### OUR BUREAU OF INFORMATION.

### Rules for Correspondents.

1. Every letter must be accompanied by the coupon to be cut from the back cover (inside page) of The Hospital, current issue, and must contain the name and address of the correspondent with pseudonym for publication if desired. Replies by post cannot be given, save under exceptional circumstances at the Editor's discretion.
2. Letters from Approved Homes in reply to special needs published in the Bureau should state terms and full particulars, and be sent prepaid under cover to the Editor of the Bureau with name written across coupon for identification.
3. Proprietors of Homes which have not yet been entered on the List of Approved Homes, but have spare accommodation likely to suit special needs, are invited to write for an application form for registration. The fee for registration, which includes two announcements of the Home in the Bureau and other privileges, is 10s.
4. All communications to be addressed to the Editor of The Hospital, 28 Southampton Street, Strand, London, W.C., and marked "Bureau of Information."

### *THE SICK AND DISTRESSED.*

**The Editor is prepared to make known without charge the needs of any who may be sick or in difficulties, and to guide them in making choice of Homes for treatment or convalescence. Reduced terms can often be arranged with Homes on the Approved List for those unable to pay full fees. Queries are specially invited from those who are engaged in any kind of philanthropical work. A new edition of the leaflet describing the purposes of the Bureau of Information is now ready and will be sent free of charge on application.**

### Help for Phthisical Case Required.

In our issues of June 7 and May 24 we drew attention to the urgent need for assistance in which this case stands. We regret to inform our readers that so far the help which is so necessary has not been rendered, and meanwhile the condition of the patient is becoming rapidly worse; for their information we briefly restate the circumstances. The patient is a young woman suffering from advanced phthisis; some time ago she was deserted by her husband, and gained her living in domestic service until overtaken by the disease. She is at present living under very unsuitable conditions in a small cottage near Shrewsbury with her parents, who are extremely poor and quite unable to provide for her needs; moreover, they have three other children living with them and dependent upon them. Owing to the advanced state of the disease, attempts to obtain sanatorium treatment for her have failed. In a letter to a friend she has again appealed for help in order that she may be enabled to get a change of air. Whilst it may be contended that her admission to a Poor-Law infirmary could easily be procured, no one can fail to sympathise with her dread of, and objection to, this proposal, and it is sometimes impossible to argue down the prejudice against Poor-Law relief which possesses the poor. We therefore once more appeal to our readers for such assistance as they can render this very deserving case. Replies should be addressed, "H. E. B.," 160B.

### Home Required.

An inexpensive home, as near the S.W. district of London as possible, is required for a boy, nine months of age, who has recently been discharged from hospital, where he has been treated for rickets. Will any of our Approved Homes able to take such a case reply to "E. G.," 161B?

### Home for Epileptic.

At the Home for Epileptics, Maghull, near Liverpool, women are received in the third-class division for a weekly payment of 10s. 6d. Owing to the numerous demands made upon this institution the period of waiting is sometimes very long. Application should be made to the Hon. Secretary, 2 Exchange Street East, Liverpool. Should you not succeed in securing admission for your patient we will gladly issue an announcement in these columns if you will say how much could be paid toward her maintenance.—"M. P."

### *INSTITUTIONAL FACTS AND FIGURES.*

### The Electricity and Water Supply of a Sanatorium.

In hospitals and sanatoria situate in outlying parts of the country the problems of water supply and illumination require careful consideration, for the fact that no central sources of supply are sufficiently near at hand with which these institutions can be connected renders it necessary that they should be in a position to provide for themselves. The following account, which we have received from a correspondent, is an interesting illustration of the manner in which these two important questions are dealt with at a sanatorium of twenty-four beds. It is convenient to deal with these questions side by side, for in the generation of light and the pumping of water power is required, and for purposes of economy this must be derived from a central source. Electricity has been chosen as the illuminating agent, for it is the most convenient form of illuminant, and, moreover, electric current lends itself to the distribution of power required for the machinery in use.

#### Description of Plant.

The plant at this sanatorium for generating electricity and pumping water consists of a steam boiler, a 6½ h.-p. steam engine, a dynamo, and two motor pumps. The boiler supplies steam to the laundry in addition, and, though this is essential for the purposes of economy, the arrangement makes it somewhat difficult to judge accurately the cost of electricity and water. The whole cost of the plant, including laundry shafting, pumps, cables, stores, etc., was £1,549. This at first sight appears excessive, but allowance is made in the equipment for future enlargement of the sanatorium.

#### Water-Supply—Well Water.

The household water is derived from two wells, 159 feet and 163 feet deep respectively, which is conveyed to storage tanks of 2,000 gallons capacity at the top of the building by electrically driven pumps. The average daily consumption is 3,500 gallons.

#### Rain-Water.

Rain-water is also stored exclusively for the purposes of the laundry and for feeding the boiler. It is collected in underground tanks of 30,000 gallons capacity, and from these conveyed to cisterns over the laundry The average weekly consumption for both these purposes is 3,500 gallons. A filter is connected with the rain-water tank, so that the water can be used for other purposes should the need arise.

#### Cost of Maintenance.

The average number of units of current consumed yearly is 4,000, 2,190 of which are used for lighting purposes and 1,810 for pumping water. The cost of production is based on the following figures: Engineer's wages, £91 9s. 8d.; fuel for boiler (coke), £60; oil, £6 11s.; repairs, wear and tear, £35 2s. 8d.; renewal of lamps, £4 19s. 6d.; total £198 2s. 10d. Allowing for the proportion of engineer's wages and of cost of fuel chargeable to laundry and other departments, the total cost of the water-supply may be estimated at about £57 a year, or 9d. per 1,000 gallons, as against 11d. charged by the water company, and the cost of light at

£69 a year. In these figures, though allowance is made for wear and tear of plant, interest on capital outlay has not been taken into consideration.

CONCLUSIONS.

It is difficult to form a really fair estimate of costs at the present stage, because provision has been made for future extensions, and the plant is therefore not used to its fullest capacity; but, taking into consideration these circumstances and the present small size of the institution, the figures given appear to be very satisfactory, and should prove suggestive to institutional workers.

[Further communications upon this subject are invited.—ED.]

### *THE INSTITUTIONAL ARTIFICER.*

**QUESTIONS FOR JUNE.**

**1. Describe and, if possible, give a sketch or photograph of any home-made piece of hospital furniture or apparatus you have designed, or which you have found to serve a useful purpose at your institution.**

**2. Describe any defects you have found n your heating and hot-water system, and the steps you have taken to remedy them.**

*NOTE.—A minimum payment of FIVE SHILLINGS will be made for each descriptive note based upon the foregoing questions which is accepted for publication.*

RULES.

The following rules must be observed:—

1. Contributions must be written on one side of the paper. They must bear the name and address of the sender and be accompanied by coupon to be cut from the back cover (inside page) of the current issue of THE HOSPITAL. A pseudonym must be chosen if the name is not to be published.

2. They must be addressed to the Editor of THE HOSPITAL, 28 & 29 Southampton Street, Strand, London, W.C., so as to reach him before the end of the month, and must be marked in the left-hand corner "Institutional Artificer."

### Water Mingler.

AMONGST the many excellent fittings at the Essex and Colchester new County Asylum is a very useful device patented by Messrs. A. Emmanuel and Sons, Ltd. This is a water mingler, an illustration of which is given herewith. It consists of a gun-metal box with inlets at the side for hot and cold water. An ingenious arrangement of valves excludes the flow of hot water until a certain amount of cold has been admitted, and it is possible to adjust the temperature of the mingled water to a nicety by consulting the thermometer which is fixed in front of the case. The mingler comprises a series of bafflers inside the box, which ensure a perfect mixing of the hot and cold water, and one inlet is fitted with a back-pressure valve, which comes into operation automatically should the pressures of water become unequal. By a concealed valve at the top of the box the amount of cold water admitted can be regulated independently of the mixed supply. The device will be welcomed by institutional workers who have had experience of the trouble which arises in the use of some water minglers owing to inequality of pressures of hot and cold water.

### *THE INSTITUTIONAL HOUSEKEEPER.*

### Laundry Materials.

THOSE responsible for the management of institutional laundries will be interested to learn that the United Alkali Company, Ltd., of Greenbank Works, St. Helens, Lancashire, have issued a Laundry Guide, as well as several instructive pamphlets dealing with the question of scientific washing preparations. Amongst the many excellent preparations placed on the market by the company is "Pearl Dust," which is especially recommended for the treatment of linen and cotton goods; soap is largely dispensed with in its use, and its value may be judged by the excellence of colour of the articles treated with it. "Marvelite," another speciality, is a rapid and effective cleansing agent, especially suitable for the treatment of silks, flannels, woollen goods, and cashmeres. The preparation has no harmful effects upon the fabrics, its softening influence avoids shrinkage, and economy in the use of soap is another advantage. The economical management of an institutional laundry depends to no small degree upon a knowledge of the materials most suitable for the work, and a perusal of the Guide referred to should prove instructive and helpful in this direction.

### *EMPLOYMENT AND TRAINING.*

### Qualifications of a Health Visitor.

YOU are, as a fully-trained nurse, quite eligible for the position of health visitor; the possession of the C.M.B. diploma, in addition to your certificate in general nursing, gives you an advantage. Either of these qualifications is acceptable in London, the only place at present where the appointment of health visitor has legal recognition and her qualifications are defined. A large number of health visitors are employed in the provinces, more especially in those areas of local government where the Notification of Births Act of 1907 has been adopted. Dual appointments of health visitor and sanitary inspector are sometimes made, and to qualify for these you must obtain the certificate of the Sanitary Inspectors' Examination Board, particulars of which may be had on application to the Secretary, 1 Adelaide Buildings, London Bridge, E.C. It is by no means easy to obtain these appointments, as competition is very keen. In certain areas the work of the health visitor and school nurse is done through the agency of the County Nursing Associations, and in some towns the local education committees make arrangements with the District Nursing Institution for the supply of nurses for school inspection. We fear that the only means you can adopt in order to secure a post is to study the advertisements which appear in the nursing papers and the local government and municipal journals from time to time.

SCHOOL MOTHERS.

The work of school mothers is promoted by the Association of Infant Consultations and School Mothers, 4 Tavistock Square, London, W.C.—"M. W."

### How to Become a Nurse.

THERE are few general hospitals which accept probationers under the age of 22, but as you say that you have turned 21 years of age, there is no reason why you should not commence to seek a suitable training school. You will find the fullest information upon this subject and a list of training schools in the book "How to Become a Nurse," published by the Scientific Press, Ltd., 28 and 29 Southampton Street, Strand, W.C., price 2s. 3d. post free.—"E. D. P."

### Infirmary Maid.

A COUPON should be enclosed with each inquiry; we cannot give postal replies. We can only advise you to study the advertisements which appear in the Nursing and Poor-Law journals from time to time, and reply to them; an alternative is to advertise your requirement.—"L. E. S."

### *EDITOR'S LETTER-BOX.*

MISS CONSTANCE BURTON. — The Editor is indebted for application and testimonials forwarded, which are being dealt with.

NURSE A. D. JARVIS.—We are very glad to hear that the suggestions made through the columns of the Bureau some time ago were instrumental in helping you to obtain a pension for your husband from the Royal Hospital for Incurables, Putney.

THE Editor will be glad to receive correspondence and to consider contributions upon all subjects relating to institutional work, and affecting the welfare of institutional officers.

### Editor's Notices.

**Contributions :**

Contributions should be written, or preferably typed, on one side of the paper only, and all articles sent in are accepted upon the distinct understanding that they are forwarded to The Hospital only.

The Editor cannot undertake to return MSS. not used, but when a stamped directed envelope is enclosed the MSS. may be returned if a special request is made.

Accepted articles and paragraphs of news will be paid for after publication at the scale rate.

**Address :**

To prevent delay all contributions and letters on editorial business must be addressed exclusively to the Editor, "The Hospital," 29 Southampton Street, Strand, London, W.C. It is important that this regulation shall be strictly observed.

**Correspondence :**

Correspondence on all subjects is invited. The name and address of correspondents must be given as a guarantee of good faith, but not necessarily for publication.

**Special Articles :**

Special articles are invited, and questions, inquiries, and paragraphs upon all matters relating to the work, administration, and management of general, special, mental, fever, cottage and convalescent hospitals, sanatoria, homes, institutions, societies and organisations for the treatment or care of the sick, injured and dependants of all classes. Every matter affecting the interests and welfare of the staff of all grades working in these institutions will receive special consideration.

**Administrative Medicine, Research and Items of News :**

Special terms will be paid for approved articles on subjects in this wide field by experts who have made some section of it their special study and interest. We wish to give prominence to every movement tending to advance accuracy of diagnosis, efficiency in treatment, and the development of modern methods to eradicate disease and promote the welfare of the suffering.

**A Bureau of Information :**

We invite inquiries and applications for information and help in providing for that numerous class of sufferers who require special care or house-room which they cannot obtain in their own homes or secure for themselves. **We cannot, however, prescribe, or recommend practitioners.**

**Books for Review :**

Publishers are particularly requested to send advance proofs of any new books of importance whenever possible, as the Editor has made arrangements to publish immediate reviews on a new plan.

**Photographs, Plans, Blocks, and Illustrations :**

It is requested that wherever possible MSS. may be accompanied by illustrations in any of the above forms. The name of the sender and of the article to which it belongs should be written on the back of each photograph, drawing, or block for purposes of identification.

**Local Papers :**

Newspapers containing reports or news paragraphs should be marked and addressed to the Sub-Editor.

**The Coupon System :**

A coupon will be found at the bottom of the third inside page of the cover each week. This coupon must be attached to every question or inquiry to which an answer is desired in The Hospital.

### Interesting Facts.

**For the Institutional Worker:**

This Special Supplement affords exceptional facilities to those seeking appointments in any grade or department of the Institutional, Health, and Sanitary Services. In this section a large number of vacancies in Hospitals, Infirmaries, and Institutions of every description throughout the United Kingdom are announced every week. Should you not find a post suited to your needs we would remind you that a column is reserved to those seeking re-engagement, and an announcement of twenty-one words, costing 1s., in this portion of the Journal will enable you to bring your requirements prominently before the Executive Officials of every class of Institution, as well as those responsible for Poor-Law and Municipal administration.

**For Institution Authorities:**

The officials controlling institutions will also find The Hospital of the greatest practical utility when a vacancy occurs upon their staffs, as an announcement in its columns secures the widest publicity among those trained to Institutional Work, and through its agency it is possible to obtain the most efficient officers in every section of Institutional Life. In these circumstances particular attention is drawn to the fact that Special Rates are allowed to those Institutions that agree to send all their public notices for insertion in this section of The Hospital.

### Manager's Notices.

**Letters relating to the publication, sale, and advertising departments of "The Hospital" must be addressed to The Manager, "The Hospital," The Hospital Building, 28 and 29 Southampton Street, London, W.C.**

**Subscriptions** may begin at any time, and are payable in advance. Cheques and Post-Office Orders should be crossed London County and Westminster Bank, Covent Garden Branch, and made payable to the Manager of The Hospital.

Rates of Subscription (payable in advance).

UNITED KINGDOM.

| | | |
|---|---|---|
| Three Months (including Postage) | | 2s. 0d. |
| Six Months | do. | 4s. 0d. |
| Twelve Months | do. | 6s. 6d. |

FOREIGN AND COLONIAL.

| | | |
|---|---|---|
| Three Months (including Postage) | | 2s. 6d. |
| Six Months | do. | 5s. 0d. |
| Twelve Months | do. | 8s. 8d. |

**Advertisements :**

To ensure insertion, all advertisements for The Hospital must reach the Manager not later than Wednesday morning in each week. For scale of charges see pages v. and 4.

**Remittances :**

**It is especially requested that remittances be made by Cheque or Postal Order, and in halfpenny stamps only when the amount is under sixpence.**

### HANDBOOKS

### For TRAINED WORKERS

POCKET-GUIDE SP SERIES

1/- net. 1/1 Post free.

**Bound in Limp Cloth.**
**Suitable Size for the Pocket.**

The works which comprise the S.P. Pocket Guide Series are intended for ready reference, consequently the aim of the Publishers has been to make them as concise and lucid as possible. Each book is written by an expert on the subject of which it treats, and the utmost reliance therefore can be placed in the information it imparts.

**Essentials of Fever Nursing.** By LYTTON MAITLAND, M.D. (Lond.), M.B., B.S., D.P.H. (Camb.).

**Manual and Atlas of Swedish Exercises.** (*With over 60 Illustrations.*) By THOMAS D. LUKE, M.D., F.R.C.S.

**Bandaging Made Easy.** (*Illustrated with over 90 Diagrams.*) By M. HOSKING, Sister-in-Charge, Tredegar House, Bow, E.

**How to Write and Read Prescriptions.** By LYTTON MAITLAND, M.D. (Lond.), M.B., B.S., D.P.H. (Camb.).

**Principal Drugs and their Uses.** By A PHARMACIST.

**Asepsis and How to Secure It.** By H. W. CARSON, F.R.C.S.

**Treatment after Operations.** *Rules for Nursing after General and Special Operations.* By MARY WILES.

**The Nurse's Duties before and during Operations.** By MARGARET FOX, Matron, Prince of Wales's General Hospital, Tottenham.

**Other works, in amplification of this Series, will be announced in due course.**

*Published by*

**The SCIENTIFIC PRESS, Ltd.**

**28/29 Southampton Street, STRAND, LONDON, W.C.**

## Figures and Tables

**Figure f1:**